# Comparison of In‐Gel and SP3 Based Sample Preparation Protocols for LC‐MS/MS Based Proteomics

**DOI:** 10.1002/pmic.70123

**Published:** 2026-03-29

**Authors:** Luisa Schwarzmüller, Dario Frey, Piotr Zadora, Martin Schneider, Stefan Wiemann, Dominic Helm

**Affiliations:** ^1^ Proteomics Core Facility German Cancer Research Center (DKFZ) Heidelberg Germany; ^2^ Division Molecular Genome Analysis German Cancer Research Center (DKFZ) Heidelberg Germany; ^3^ Faculty of Biosciences University Heidelberg Heidelberg Germany; ^4^ Proteomics Core Facility The University of Texas MD Anderson Cancer Center Houston Texas USA

**Keywords:** bottom‐up proteomics, in‐gel digestion, protein and peptide level fractionation, sample preparation, single‐pot Solid‐Phase Enhanced Sample preparation (SP3)

## Abstract

Sensitivity, robustness, and reproducibility of sample preparation are main determinants of data quality in bottom‐up mass spectrometry‐based proteomics. To this end, in‐gel protein clean‐up and digestion has been used for decades and is characterized by its robustness and compatibility with harsh lysis conditions. Single‐pot solid‐phase‐enhanced sample preparation (SP3) has gained substantial popularity recently and has been widely adapted as a standard workflow often replacing in‐gel digestion‐based workflows. Noteworthy, until today no direct comparison between the two workflows has been conducted. Here, we performed a systematic comparison of in‐gel and SP3 based sample preparation workflows assessing sensitivity, robustness, reproducibility, and fractionation possibilities on human cellular lysates and blood plasma. Both methods performed similarly regarding number of identified proteins, however, showed specific biases. SP3 outperformed the in‐gel workflow regarding higher sensitivity when handling limited sample amounts, especially below 5 µg of input material. In contrast, in‐gel sample preparation was superior in the identification of low molecular weight proteins. In conclusion, while SP3 is indeed the state‐of‐the‐art proteomics sample preparation method, in‐gel digestion can deliver competitive and complementary results and still has advantages in some applications, such as measurement of small proteins or when there is a need for protein‐level separation, e. g. in plasma samples.

**Abbreviations**
CIF: Carboxylate‐Modified Magnetic Bead (CMMB)‐Based Isopropanol Gradient Peptide Fractionation
DDA: Data‐dependent acquisition
DIA: Data‐independent acquisition
MW: Molecular weight
RP: high pH reversed‐phase
SDS‐PAGE: Sodium Dodecyl Sulfate—Polyacrylamide Gel Electrophoresis
SP3: Single‐pot solid‐phase‐enhanced sample preparation

## 1 Introduction

Mass spectrometry (MS)‐based proteomics has become a fundamental tool in biomedical research [1, 2, 3], owing to improved technologies, methods, and analysis software tools [4, 5, 6, 7]. Specifically, the number of detectable proteins from a single measurement has been vastly increased in the last years through increased yields during sample preparation workflows [4, 5, 6] and the development of faster and more sensitive mass spectrometry instrumentation.

Despite these impressive advances, some fundamentals have remained unchanged: sample clean‐up and proteolytic digestion are required prior to bottom‐up LC‐MS/MS proteome analysis. For successful extraction of proteins from primary material or cultivated cells, detergents, such as sodium dodecyl sulfate (SDS), Tween or NP‐40, chaotropes, such as guanidine or urea, and high concentrations of salts are used. If not removed by efficient sample clean‐up, these agents interfere with the mass spectrometric measurement. Furthermore, in bottom‐up proteomics proteins are proteolytically digested to generate peptides in a compatible size window for ionization and mass detection, and a buffer exchange needs to be performed in order to create suitable conditions for the protease to be functional [7].

A variety of different methods for protein sample clean‐up and subsequent digestion have been developed. A tryptic digest performed in polyacrylamide gels following SDS‐PAGE (Sodium Dodecyl Sulfate—Polyacrylamide Gel Electrophoresis) is the most traditional method for proteomic sample preparation [8, 9]. Identification of proteins from polyacrylamide gels was introduced already back in the 1980s [10], first for Edman sequencing [11] and since 1993 for mass spectrometry base analysis [12, 13, 14]. In‐gel‐digestion for proteomics sample preparation has remained popular until today for various reasons, especially among core service infrastructures: I) It allows for the separation of complex protein mixtures by gel electrophoresis and thereby offers the possibility to analyze single gel bands [15]. II) The method is compatible with a wide range of lysis conditions [16]. III) Proteins in gel are stable and can be stored for years [17]. IV) Separating proteins by molecular weight offers a protein‐level sample fractionation method for a deeper analysis of the proteome [18, 19].

To reduce sample loss and thus increase sensitivity, several “one‐pot” MS sample preparation methods have been developed in the past years [5, 20, 21, 22]. Among these, the single‐pot, solid‐phase‐enhanced sample preparation (SP3) [6] has become particularly popular in the proteomics community as it combines some advantages of in‐gel, i.e buffer compatibility [6, 23], and one‐pot methods, i.e. higher throughput and reduced sample loss. Furthermore, the SP3 protocol is relatively easy to adapt and can be readily automated [24, 25], thus enabling high throughput sample preparation, essential e.g., for laboratories with a high sample load as in core infrastructures or for large cohort studies. Additionally, on‐bead peptide level fractionation can be performed as an additional step to further increase the proteome coverage [26].

Several studies have been performed comparing different proteomics sample preparation methods and it has been demonstrated that the choice of sample preparation method impacts the results of an experiment [27, 28, 29, 30, 31]. Hayoun et al. published a comparison of sample preparation methods, including in‐gel and SP3, for fast proteotyping of microorganisms [32]. However, to the best of our knowledge, no study has directly compared in‐gel and SP3 protocols in the context of classical proteomics.

Here, we systematically compared in‐gel sample preparation with the SP3 approach using a human tumor cell line and a plasma sample. We digested different input amounts of proteins from the cell lysate, prepared samples with in‐gel and SP3 protocols, respectively, and compared the sensitivity of the methods. Both methods delivered comparable numbers of identified proteins when applied to higher protein input amounts, however SP3 demonstrated higher sensitivity when handling limited sample amounts. Exploring fractionation possibilities of both methods revealed that protein level in‐gel fractionation is advantageous for identifying low molecular weight proteins. This became evident especially when applied to a human plasma sample, characterized by a high dynamic range of protein abundance, where in‐gel protein level fractionation allowed for increased identification compared to peptide level fractionation workflows and allowed the detection of low‐abundant and small proteins.

## 2 Results and Discussion

### 2.1 In‐Gel and SP3 Perform Similarly Regarding Protein Identification and Quantification

**FIGURE 1:**
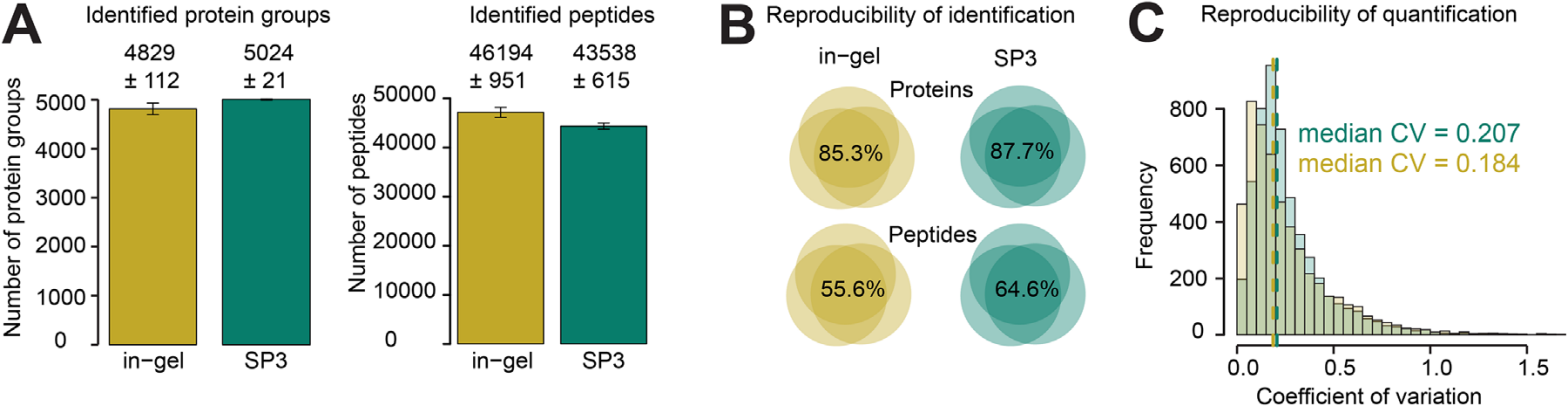
Qualitative comparison of in‐gel and SP3 full proteome sample preparation. Protein clean‐up and digestion of MCF7 lysate (20 µg protein input) was manually performed by in‐gel and SP3 protocols, respectively (*n*=3). MS measurements were performed in DDA mode for a total analysis time of 120 min. Raw data were searched using MaxQuant. (A) Number of identified protein groups and peptides. Mean and standard deviation are indicated. (B) Reproducibility of protein group and peptide identification between triplicates. Percentages of proteins/peptides that were reproducibly identified in all replicates are indicated. (C) The coefficient of variation (CV) was calculated based on the log2‐transformed raw intensities as the standard deviation divided by the mean.

To compare the performance of two commonly used state‐of‐the‐art sample preparation methods for mass spectrometry‐based proteomics, we used lysates of the human luminal A breast cancer cell line MCF7. We performed protein clean‐up and tryptic digestion of 20 µg protein, following either in‐gel or SP3 strategies. Mass spectrometry‐based analysis using data‐dependent acquisition (DDA) and data analysis using MaxQuant revealed similar numbers of identified proteins and peptides for both methods (Figure 1A), with a slightly higher number of peptides being identified by the in‐gel approach. The reproducibility of protein and peptide identification between the replicates (*n* = 3) was comparable between both methods (Figure 1B).

While the numbers of identified proteins and peptides were similar, the chromatograms of the measurements showed differences in overall intensities (Figure S1A). To systematically evaluate this observation, we analyzed different injection amounts of the same digestion and summed up the MS1 intensities to get a measure of overall sample intensity. This revealed, on average, 18% lower signal intensities for the in‐gel workflow compared to SP3, suggesting differences in protein/peptide losses during sample preparation (Figure S1B). To quantitatively assess the recovery of peptides from in‐gel and SP3 digestion, we next compared the chromatograms of “theoretical” 500 ng peptides from either sample preparation method to a commercial complex human tryptic digest (purchased from ThermoFisher, Figure S1C). “Theoretical” 500 ng of peptide were calculated based on the assumption that both processes resulted in zero losses (e g. 10 µg of protein would yield 10 µg of peptide). Analyzing a fraction (i e. 1/20) of the samples from in‐gel digestion or SP3 would correspond to “theoretical” 500 ng of peptide on column. These measurements were compared to 500 ng of peptide on column of the commercial tryptic digest as reference. This revealed that the MS1 total ion current (TIC) of the samples prepared by either method was more than two‐fold lower compared to the commercial standard sample, indicating significant sample loss with both methods. To precisely quantify the extent of sample loss, we then established a linear calibration curve by injecting known amounts of the commercial peptides, ranging from 50 ng to 1000 ng (Figure S1C). Using the calibration curve, the actual peptide amounts measured in the SP3 and in‐gel samples revealed that in‐gel digestion recovers only 18%–22% of peptides, while SP3 performs better and recovers 20%‐28% of the peptides (Figure S1C). The digestion efficiency, assessed by missed cleavage sites and peptide length, appeared to be higher for the commercial HeLa digest (Figure S1D), suggesting that the tryptic digestion of our samples was slightly less efficient compared to the commercial sample. This difference was, however, not large enough to explain the more than 70% MS1 intensity loss of both SP3 and in‐gel protocols, suggesting that sample loss must have occurred at earlier stages. A study by Johnston et al. [33] has shown that protein recovery with SP3 is substantially influenced by the protein concentration during the binding step to the magnetic beads, which is not equal to the protein concentration of the lysate. In our study, we set the volume to 200 µL (protein concentration to 0.1 µg/µL) during the bead binding step. Thus, the estimated peptide recovery of 20%–28% (Figure S1C) agreed with the observations of Johnston et al. [33]. In conclusion, compared to the in‐gel approach, we observed a better protein recovery with SP3, which could potentially be further improved by reducing the volume, thus increasing the protein concentration in the protein binding step.

Next, we investigated the reproducibility of protein quantification with in‐gel and SP3 protocols. Indeed, the coefficients of variation (CV) of the log2‐transformed raw protein intensities (unnormalized intensity values provided by MaxQuant in proteinGroups.txt file) were similar between the technical replicates of both sample preparation methods, with a median of 18.4% and 20.7%, respectively (Figure 1C). High correlation between the protein intensities measured in replicates within the same method was observed with Pearson correlation coefficients > 0.95 (*R* ^2^ > 0.91) for both workflows. The Pearson correlation coefficients were lower (around 0.88; *R* ^2^ around 0.77, Figure S1E) when comparing the protein intensities obtained with the two different methods. In conclusion, both sample preparation methods resulted in comparable numbers of identified peptides and proteins with similar reproducibility of both, identification as well as quantification when using 20 µg of starting material. However, some qualitative and quantitative differences between protein groups identified were observed with the in‐gel and SP3 methods, which we focused on in the next steps.

### 2.2 In‐Gel and SP3 Differ in the Detection of Specific Peptide and Protein Classes

**FIGURE 2:**
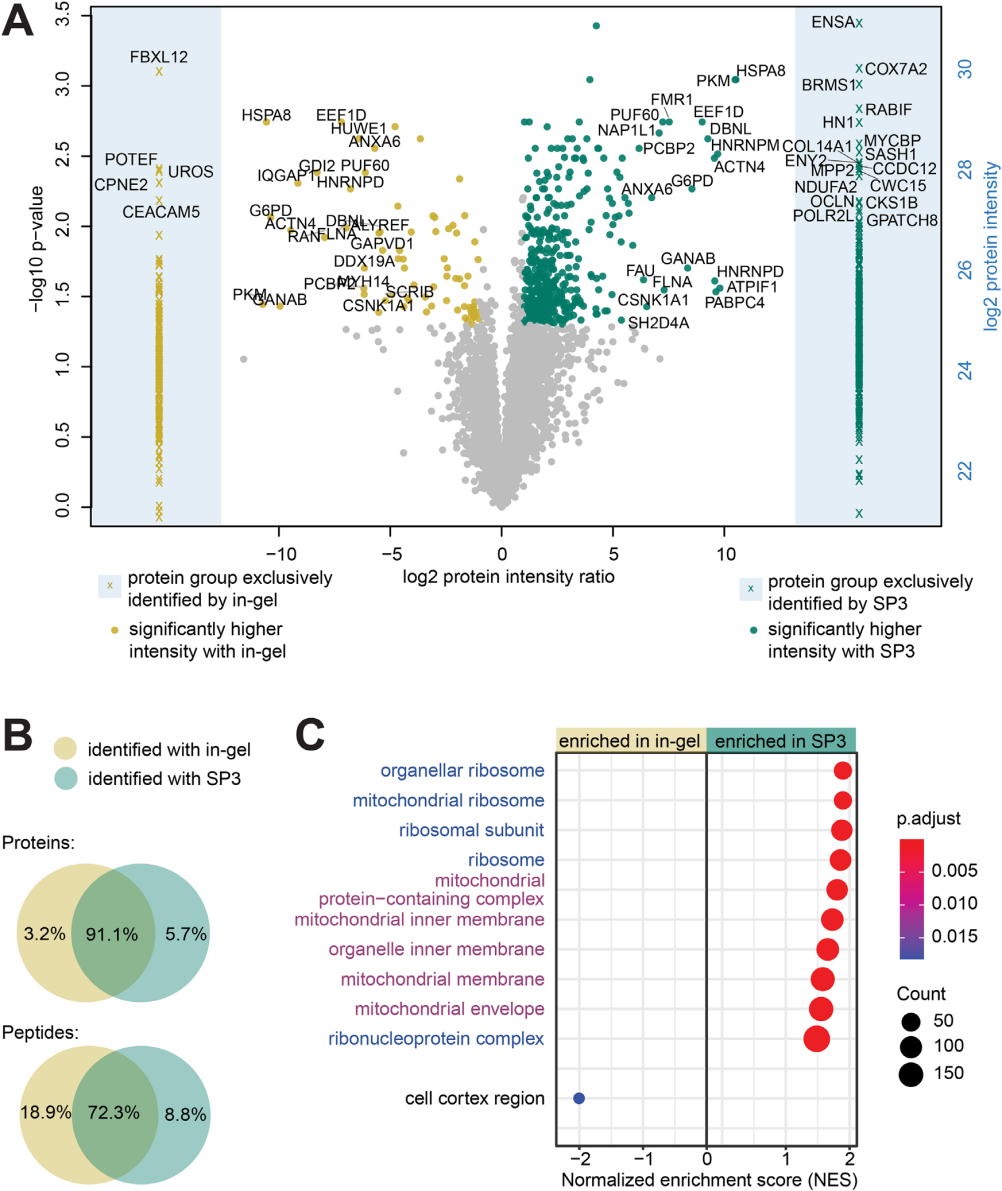
Quantitative comparison of in‐gel and SP3 full proteome sample preparation. Protein clean‐up and digestion of MCF7 lysate (20 µg protein input) was manually performed by in‐gel and SP3 protocols, respectively (*n*=3). MS measurement was performed in DDA mode for a total of 120 min LC‐MS/MS analysis time. Raw data were searched using MaxQuant. (A) Log2 fold‐change of mean SP3 intensities vs. mean in‐gel intensities and Benjamini‐Hochberg‐adjusted *p*‐values are presented. Significantly differentially quantified protein groups (adjusted *p*‐value < 0.05, absolute log2 FC > 1) are highlighted and labelled with the respective gene name. Proteins identified exclusively by one sample preparation method are shown as “x” and high intense protein groups are labelled with the respective gene name. (B) Reproducibility of protein and peptide identification between the two sample preparation methods. Percentages of proteins/peptides identified by both in‐gel and SP3 methods, are indicated. (C) The gseGO function of the ClusterProfiler [46] R package was used for gene set enrichment analysis of GO‐terms (cellular compartment). Dotplot visualization of the top10 significantly enriched GO‐CC terms in SP3 and all significantly enriched GO‐CC terms in in‐gel. Related gene sets are highlighted.

To investigate quantitative differences between the two methods, obtained raw protein intensities were compared by calculating log2‐fold‐changes and adjusted p‐values (unpaired, two‐sided t‐test, Benjamini‐Hochberg adjustment, Figure 2A). This statistical analysis revealed that 160 protein groups were recovered at significantly higher intensities with the in‐gel method, while 608 protein groups showed a significantly higher intensity when using SP3. Additionally, 174 protein groups were identified exclusively in the in‐gel samples, while 310 protein groups were identified only with SP3. The identification of those exclusive proteins was not always related to low protein intensity alone but appeared to be related to the sample preparation method that was applied (Figure 2A). Overall, the overlap in protein identifications was high with 91.1% of the protein groups identified with both in‐gel and SP3 protocols, whereas only 72.3% of peptides were identified by both methods (Figure 2B). The low overlap of peptide identifications between both sample preparation methods could be a measurement artefact from a DDA method (see later section). Performing a gene set enrichment analysis (GSEA) of cellular compartment gene ontology terms (GO‐CC) of proteins detected by both methods revealed that ribosomal proteins and mitochondrial membrane proteins were recovered significantly better with the SP3 protocol, while proteins with annotation “cell cortex region” were recovered more efficiently using the in‐gel method (Figure 2C). In conclusion, proteomics sample preparation with the in‐gel or SP3 protocols, respectively can lead to more efficient recovery of different protein classes. Potentially, the heating of lysates to 70°C in the presence of LDS buffer which was done in preparation for the in‐gel workflow, might explain the differential protein extraction.

### 2.3 SP3 is More Robust than In‐Gel for Low Amounts of Starting Material

**FIGURE 3:**
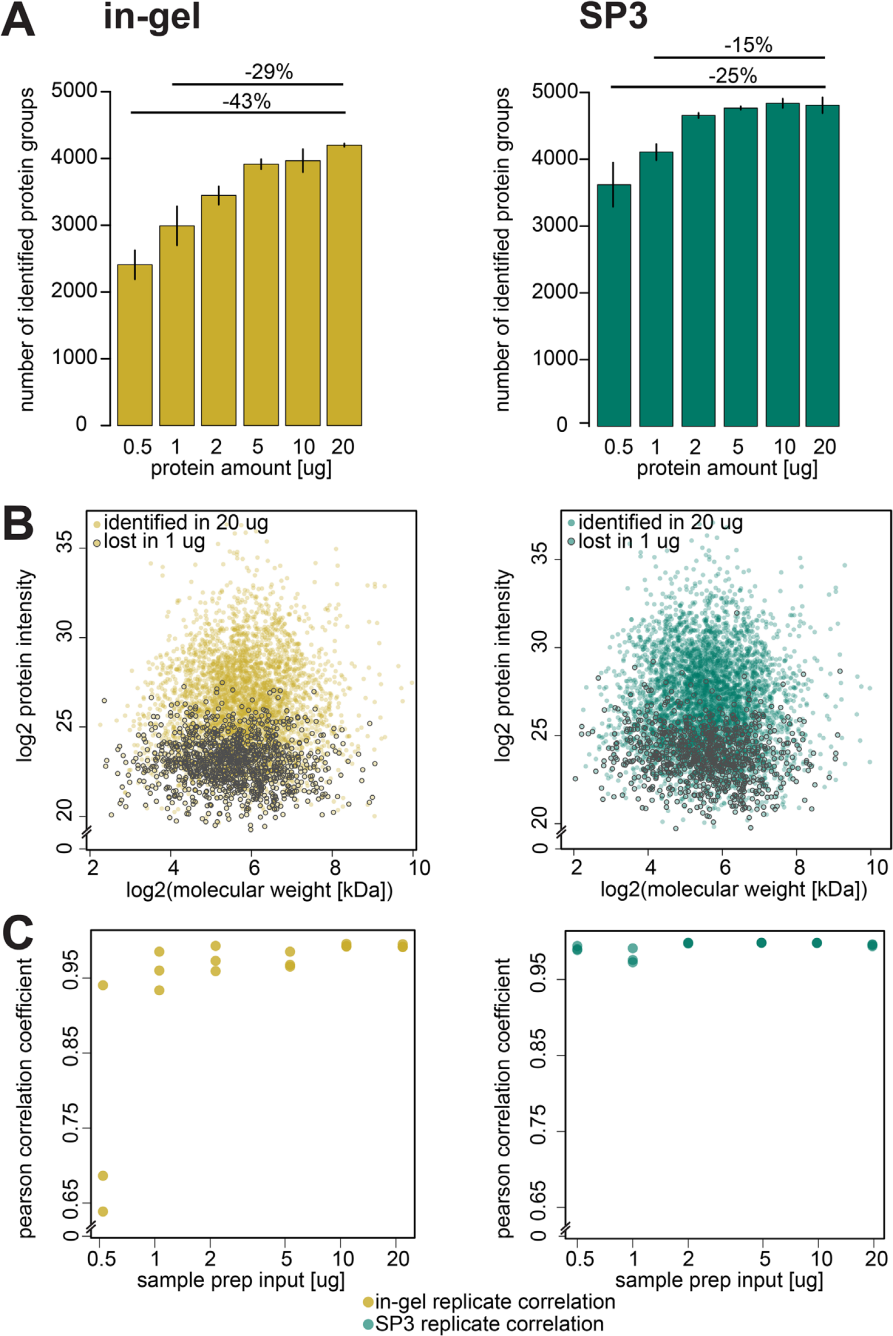
Comparison of in‐gel and SP3 workflows with regards to sensitivity. Protein clean‐up and digestion of MCF7 lysate (20 µg – 0.5 µg protein input) was manually performed by in‐gel and SP3 protocols, respectively (*n*=3). The same theoretical amounts of peptides (0.5 µg) was injected for each sample for MS measurement in DDA mode (120 min). Raw data were searched using MaxQuant. (A) Shown are the number of protein groups identified from different protein starting amounts with in‐gel or SP3, respectively. (B) The molecular weight (kDa) and log2 protein intensities are shown for all proteins identified by in‐gel or SP3, respectively, with the highest amount of starting material (20 µg). Proteins that were not identified in the low input condition (1 µg) are highlighted. (C) The Pearson correlation coefficients of protein intensities were calculated for each combination of the triplicates.

While we observed that both in‐gel and SP3 methods led to qualitatively similar results when using high protein input amounts (20 µg), limited amounts of sample are a concern, particularly when precious patient samples shall be analyzed. Thus, we next assessed the sensitivity of in‐gel and SP3 protocols with respect to lower amounts of protein starting material. To this end, a series of different starting amounts, ranging from 20 µg to 500 ng of protein input amount, was prepared with the in‐gel or SP3 methods, respectively. Then, a fraction of each sample theoretically corresponding to 500 ng of protein (assuming no loss for convenience of calculation) was subjected to LC‐MS measurement. With the in‐gel method, the numbers of identified protein groups dropped slightly already when using 10 µg or 5 µg protein as input (Figure 3A). Even lower input material amounts, i.e. 1 µg and 500 ng, led to a 29% and 43% decrease in the numbers of identified proteins, respectively. In contrast, protein identifications were stable for the SP3‐processed samples even with 2 µg of protein as input and dropped only for the 1 µg and 500 ng input by 15% and 25%, respectively (Figure 3A). These findings are in line with a previous study, where SP3 was tested for sample preparation with low µg amounts of protein [29]. With both methods, mainly lower abundant proteins were lost when lower protein input amounts were used (Figure 3B). The Pearson correlation coefficient was constantly high (> 0.95) between the replicates with SP3 sample preparation, while higher inter‐replicate variance was observed with the in‐gel experiments, especially for the 500 ng input samples (Figure 3C). We argue the difference in sensitivity is inherent in the SP3 method being a single‐pot reaction in comparison to the manual in‐gel workflow that includes multiple pipetting steps using multiple reaction tubes, which increases the number of times the sample is in contact with plastic surfaces. Even using low‐binding plastic ware will still result in a more pronounced sample loss. Therefore, SP3 appears to be more sensitive and better suited for the processing of samples with limited amounts of starting material.

### 2.4 Protein Level In‐Gel Fractionation is Superior to Peptide Level Separation for the Detection of Low Molecular Weight Proteins

**FIGURE 4:**
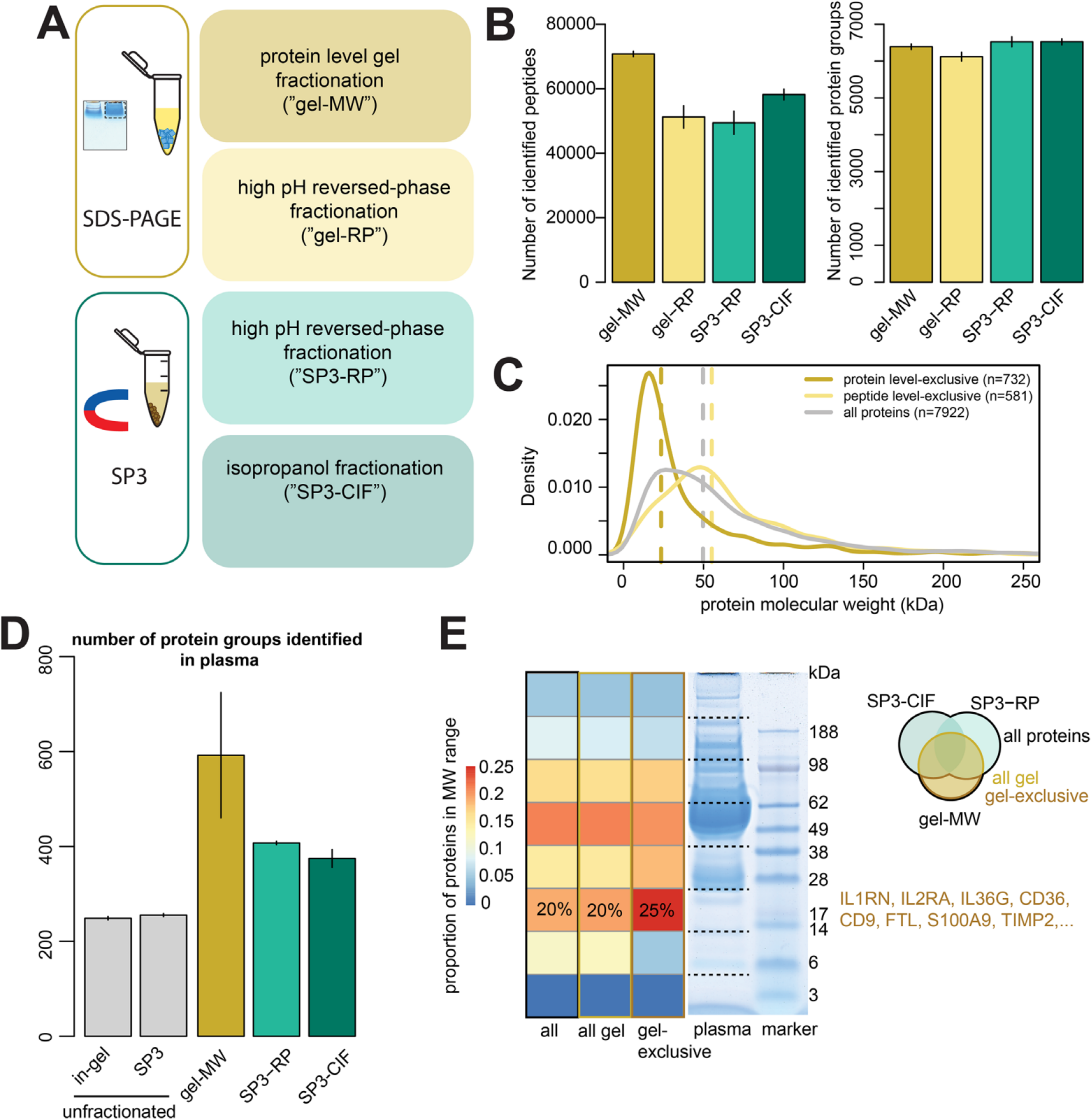
Comparison of in‐gel and SP3 sample fractionation approaches on human cell line lysate and plasma sample. Protein clean‐up and digestion of MCF7 lysate (20 µg protein input) was manually performed by in‐gel and SP3 protocols, respectively (*n*=3). MS measurement was performed in DDA mode (120 min or 90 minutes per fraction). Raw data were searched using MaxQuant with match‐between‐runs enabled for the fractions. (A) Schematic representation of the four different sample fractionation strategies. (B) The total numbers of peptides and protein groups identified. (C) The protein molecular weight (kDa) distribution of proteins identified exclusively by the protein‐level (“gel‐MW”) or peptide‐level (“gel‐RP”) in‐gel fractionation, respectively, are shown and compared to the MW distribution of all identified proteins. Respective median molecular masses are indicated by the dashed lines. (D) In‐gel and SP3 workflows were applied to a human plasma sample and the numbers of identified protein groups (*n*=3) are shown. (E) Coomassie‐stained gel of plasma sample processed with SDS‐PAGE. Cutting lines for protein‐level in‐gel fractionation (“gel‐MW”) are indicated by the dotted lines. For each molecular weight range, the proportion of all, all in‐gel, and exclusively in‐gel identified proteins was calculated, respectively. The percentages of proteins within the 13 kDa—25 kDa range are highlighted.

Sample fractionation methods based on different chemical properties can be applied to both in‐gel and SP3 workflows either by fractionation on protein level or after digestion on peptide level. Here, we compared four frequently used fractionation approaches to assess the effect on proteome coverage: i) molecular weight‐based in‐gel fractionation at the protein level (“gel‐MW”), ii) in‐gel digestion followed by high pH reversed‐phase peptide fractionation (“gel‐RP”), iii) SP3 followed by high pH reversed‐phase peptide fractionation (“SP3‐RP”), and iv) SP3 followed by Carboxylate‐Modified Magnetic Bead‐Based Isopropanol Gradient peptide Fractionation [26] (“SP3‐CIF”, Figure 4A).

The “SP3‐CIF” and “gel‐MW” fractionation methods showed the best orthogonality to the low pH hydrophobicity‐based fractionation of the LC analysis with respect to the number of identified precursors for each fraction over the retention time (Figure S2A). In contrast, but consistent with prior knowledge, reversed‐phase fractionation methods using a high pH led to identification of mostly hydrophilic peptides in the early fractions while mainly hydrophobic peptides were detected in the later fractions, in combination with both, in‐gel and SP3. Thus, these methods are not orthogonal to the low pH reversed‐phase separation of the LC system and do not optimally distribute the peptides over the whole range of the retention time and might allow for sample pooling to optimize LC‐MS time. While gel‐RP, SP3‐RP, and SP3‐CIF methods resulted in relatively uniform numbers of peptides identified in each fraction, more peptides were identified in the middle fractions of the gel‐MW approach, reflecting the higher protein density in the intermediate molecular weight fractions (Figure S2B). To estimate the effectiveness of protein separation, we counted the number of fractions each protein was identified in. Protein separation was most complete, as expected with the gel‐MW approach, resulting in most proteins being identified in only a single fraction. In contrast, with the SP3‐CIF method most proteins were found in all six fractions (Figure S2C). Also, protein sequence coverage was highest with the gel‐MW fractionation method where the identified peptides covered, on average, 25% of the protein amino acid sequence (Figure S2D). Nonetheless, all fractionation methods led to a similar increase in the number of identified proteins of 27%–32% compared to an unfractionated sample (Figure 4B, compare to Figure 1A). Interestingly, the overall dynamic range of protein intensities was however, not increased by the fractionation (Figure S2E).

Next, we investigated whether fractionation at the peptide level (“gel‐RP”) or at the protein level (“gel‐MW”), respectively, would show any specific advantages. There, protein‐level in‐gel fractionation (“gel‐MW”) outperformed the other methods regarding the numbers of identified peptides (Figure 4B, right panel). Proteins that were exclusively identified using the “gel‐MW” method were enriched for relatively small proteins with a median molecular weight of 25 kDa (Figure 4C). Physical separation of small and larger proteins in PAGE thus seems to aid the detection of low molecular weight proteins that are otherwise difficult to detect, especially in complex mixtures, potentially due to the lower number of peptides they can give rise to. We additionally conducted state‐of‐the‐art DIA measurements (*n* = 4) without offline fractionation. The DIA analysis increased significantly the overall depth of protein group identifications for in‐gel (avg. 6426 proteins) and SP3 (avg. 6924 protein groups) workflows (Figure S3A) in comparison to our initial DDA measurements without prior fractionation and on the same level to the offline fraction data. Additionally, the overlap of peptide identifications between the two sample preparation methods on also increased to 80.1% (Figure S3B). Underscoring that, in general, nowadays pre‐fractionation methods can be skipped when using DIA over DDA acquisition. However, the relative advantage of protein‑level fractionation for detecting small proteins remained unchanged, indicating that the observed benefit is inherent to the in‐gel workflow (Figure S3C). Exclusively identified peptide sequences, on the other hand, seem to differ in the length distribution between both methods. While SP3 digestion exclusive peptides tend to be shorter in length, in‐gel digestion seems to result in longer peptides exclusive to this workflow (Figure S3D). Noteworthy, performing an extensive PTM search for both DIA datasets, revealed that none of the workflows introduced major modification artifacts apart from oxidation of Methionine which was significantly higher for the manual in‐gel digestion workflow. This observation could be attributed to the increased number of transfer steps (i e air exposure) in the in‐gel digestion workflow compared to the single‐pot workflow of SP3. (Figure S4).

The analysis of liquid biopsies from blood plasma has recently gained substantial interest, especially in the field of clinical proteomics. However, given the unfavorable distribution of protein abundances within these samples, with proteins like albumin and immunoglobulins making up almost 90% of it [34], we reasoned that this sample type could benefit most from protein level separation. Furthermore, enhanced detection of small proteins would be also advantageous for the mass spectrometry‐based analysis of plasma samples, since many proteins of interest (e g. interleukins and other signaling proteins) are of low molecular weight and their detection is often hampered by the presence of highly‐abundant, larger proteins (e.g. human serum albumin) [35, 36]. To test this hypothesis, we applied the in‐gel and SP3 workflows to a commercial human plasma sample and observed that protein‐level fractionation using in‐gel digestion outperformed the unfractionated samples, as well as both SP3‐based peptide level fractionation approaches (Figure 4D). A closer look at the proteins exclusively identified by protein‐level fractionation (via “gel‐MW”) revealed that the main advantage indeed lies in the detection of proteins in the range of 13–25 kDa (Figure 4E). Among these proteins are, for instance, interleukin receptor chains (IL1RN, IL2RA), cell surface markers (CD9, CD36) and known clinical biomarkers such as S100A9, which is a plasma biomarker of acute inflammation and other malignancies [37, 38, 39]. Further examples are TIMP2 which is used to predict the risk for developing acute kidney injury in surgery patients [40] and FTL, a plasma biomarker for glioblastoma [41, 42]. It thus seems that in‐gel and SP3 protocols have both advantages and disadvantages and selecting the best sample preparation method depends on the biomedical question and the particular application. While likely unsuited for clinical applications, since high‐throughput implementations of in‐gel protein‐level fractionation are not feasible in a straightforward fashion, the method is worth considering for smaller projects that aim to identify and quantify small proteins from complex samples. Our results support this view, while also aligning with the growing popularity of high‐throughput bead‐based sample preparation methods—such as the Seer Proteograph workflow [43], the Biognosys P2 enrichment workflow, and the Mag‐Net method [44] which all incorporate a separation/depletion step on protein level.

## 3 Materials and Methods

### 3.1 Cell Culture and Lysis

The luminal A breast cancer cell line MCF7 (RRID: CVCL_0031, obtained from ATCC/LGC Standards GmbH, Wesel, Germany) was cultivated in DMEM (Gibco, Cat. 11995073), supplemented with 10% FBS, 1% Pyruvate, 1% P/S, 1 uL estrogen. Cells were lysed in RIPA buffer (Thermo, Cat. 89900) supplemented with 10 mM NaF, 1 mM Na3VO4, complete EDTA‐free protease inhibitor (Roche), PhosSTOP phosphatase inhibitor (Roche), 250 U/ml Benzonase and 10 U/ml DNase. Lysates were centrifugated at 4°C and max. speed for 30 min. Protein concentration was determined by BCA assay, and the lysate was stored at −80°C. The cell line was regularly authenticated by STR profiling (Multiplexion GmbH Heidelberg, Germany) as well as tested for potential mycoplasma contamination.

### 3.2 Plasma Sample

Human plasma was obtained from Sigma–Aldrich (product number P9523) and was dissolved in 1 mL MS‐grade H_2_O. To determine the protein concentration, different volumes of plasma were subjected to SDS‐PAGE (10 min, 100 V) together with a BSA standard curve and stained with Coomassie for 3 h. The band intensities were quantified using ImageJ and the plasma protein concentration was estimated with the BSA standard curve.

### 3.3 Protein Clean‐Up and Digestion by Manual SP3 [6]

SP3 was performed with 20 µg protein of the MCF7 RIPA lysate, unless noted otherwise for a respective experiment. Volume was adjusted to 60 µL using RIPA buffer supplemented with 100 mM TEAB. For protein reduction and alkylation, TCEP and CAA were added to final concentrations of 10 mM and 40 mM, respectively. Reduction and alkylation were performed for 5 min at 95°C. Subsequently, 2 µL of paramagnetic beads (Sigma–Aldrich #45152105050250 and #65152105050250 mixed 1:1 to final concentration of 100 µg/µL) were added, followed by addition of ethanol to a final concentration of 50%. Protein precipitation and binding to the beads was performed for 20 min at room temperature at 650 rpm. Bead‐bound proteins were washed twice with 80% ethanol and once with 100% acetonitrile. After removal of acetonitrile, beads were reconstituted in 100 mM TEAB containing Trypsin in a protease:protein ratio of 1:25. Protein digestion was performed for 16 h at 37°C and 650 rpm. The peptide containing supernatent was collected and vacuum centrifuged until dry. Dried peptides were stored at −20°C until measurement.

### 3.4 SDS‐PAGE and Manual In‐Gel Digestion [8]

For SDS‐PAGE, 10 µg of protein lysate was used, unless noted otherwise for a respective experiment. 4× LSD running buffer (Thermo Fisher, #NP0007) and 10× reducing agent (Thermo Fisher, #NP0009) were added and the samples were heated to 70°C for 10 min. Samples were loaded onto NuPAGE Bis–Tris (4%–12%) gels (Life Technologies, #NP0321BOX) and placed in MES buffer (Life Technologies, #NP0002). An electric field of 20 V/cm was applied for 9 min letting proteins migrate up to 0.5 cm into the gel. Then, gels were stained in Coomassie Brilliant blue R‐250 for 3 h and the 0.5 cm long protein band was cut out. The band was further cut into 6 smaller pieces for further processing.

100 µL water was added to the gel pieces and incubated for 5 min at 37°C and 600 rpm. The supernatant was discarded and the gel pieces were washed in 100 µL 50% acetonitrile for 5 min at 37°C and 600 rpm. For reduction of disulfide‐bridges, 100 µL 10 mM DTT was added and incubated for 60 min at 56°C and 600 rpm. Subsequently, gel pieces were washed with 100 µL water as described before. For alkylation, 100 µL 55 mM IAA was added and incubated for 30 min in the dark at 25°C and 600 rpm. Gel pieces were washed three times each with water and 50% acetonitrile as described previously. To de‐hydrate the gel pieces, 100% acetonitrile was added, incubated for 1 min at room temperature and the supernatant was discarded. This step was repeated once. Gel pieces were air‐dried for 15 min. Subsequently, 15 µL of Trypsin (dissolved in 50 mM TEAB) was added to a final protease:protein ratio of 1:40. Once the Trypsin was soaked into the gel pieces, they were covered with further 50 µL of 50 mM TEAB. Digestion was performed for 16 h at 37°C and 500 rpm.

For extraction of peptides from the gel pieces, 50 µL of 50% acetonitrile with 0.1% TFA was added and incubated in the sonication bath for 5 min. The supernatant was collected in a fresh tube. This procedure was repeated with 100% acetonitrile, 0.1% TFA again 100% acetonitrile. The collected supernatants were vacuum centrifuged until dry and the dried peptides were stored at −20°C until measurement.

### 3.5 In‐Gel (Protein‐Level) Fractionation

For protein level fractionation, 20 µg protein was used as input and SDS‐PAGE was performed as described above with the alteration that the gel was run at 20 V/cm for 20 min. The gel was stained in Coomassie and the 4 cm long protein‐containing area was cut into 8 pieces of 0.5 cm each. Manual in‐gel digestion of each piece was performed as described previously with 0.25 µg Trypsin per gel piece. Following 16 h of digestion, peptide extraction was performed as described above. Peptides were vacuum centrifuged to dryness and stored at −20°C until measurement.

### 3.6 High pH Reversed‐Phase Fractionation

Digested peptides from 20 µg input material were processed either with manual SP3 or manual in‐gel digestion. The resulting peptides were fractionated using the Pierce high pH reversed‐phase peptide fractionation kit (Thermo Scientific, Cat. 84868) according to the manufacturer's instructions. Briefly, the columns were washed twice with acetonitrile and twice with 0.1% TFA (centrifuge at 5000 g for 2 min in each wash). The dried peptides were reconstituted in 300 µL of 0.1% TFA and loaded onto the column (centrifuge at 3000 g for 2 min). The peptides were washed once with water. Elution was performed with eight solutions of 0.1% Triethylamine in water with increasing amounts of acetonitrile (5%, 7.5%, 10%, 12.5%, 15%, 17.5%, 20%, and 50% v/v). The resulting fractions were vacuum centrifuged to dryness and stored at −20°C until measurement.

## 4 CIF—Carboxylate‐Modified Magnetic Bead (CMMB)‐Based Isopropanol Gradient Peptide Fractionation

SP3‐based isopropanol gradient peptide fractionation was performed according to the instructions published by Deng et al. [26]. SP3‐digested peptides from 20 µg protein input were reconstituted in 10 µL water and divided into 2×5 µL in PCR tubes. Magnetic beads were prepared as described above and 5 µL of the bead preparation was added to the peptides. Acetonitrile was added to a final concentration of 95% and peptide binding to the beads was performed for 10 min at room temperature at 650 rpm. Subsequently, the supernatant was discarded and the peptides were eluted from the beads by decreasing concentrations of isopropanol in water (90%, 85%, 80%, 75%, 70%, and 0%). Each elution step was performed twice with 50 µL of the respective isopropanol solution and the beads were pipetted up and down 15× to ensure proper mixing with the elution solution. The fractionated peptides were vacuum centrifuged to dryness and stored at −20°C until measurement.

### 4.1 LC‐MSMS Measurement

For LC‐MSMS measurement, peptides were reconstituted in MS‐grade water supplemented with 0.1% trifluoroacetic acid (TFA) and 2.5% hexafluoroisopropanol (HFIP).

After digestion, the resulting peptide amount was not determined. Instead, the peptide amount for the LC‐MS analysis was calculated based on an (theoretical) assumption that both processes resulted in no loss (e.g. 10 µg of protein would yield 10 µg of peptide). For unfractionated samples a “theoretical” 1 µg of peptide was injected for analysis. Using the Ultimate 3000 liquid chromatography (LC) system (Agilent), the peptides were separated on a 25 cm column (Waters nanoEase^TM^ BEH C18 130Å, 1.7 µm, 75 µm × 250 mm, Cat. 186008795) with a 102 min gradient (120 min total method time) of 4%–30% acetonitrile at a flow rate of 300 nL/min. Mass spectrometric analysis was performed with the Orbitrap Exploris 480 (Thermo Fisher).

For DDA measurements, MS1 scans were acquired at a resolution of 60,000, the AGC target was set to 3e6 ions and the maximum ion injection time was 45 ms. MS2 spectra were acquired for up to 1 s after each MS1 spectrum at a resolution of 15000. Precursors were isolated with an isolation window of 1.4 m/z. The AGC target was set to 1e5 and the maximum ion injection time was 22 ms. For the dilution curve measurement, theoretical 500 ng peptides were injected and the same LC and MS1 settings were applied. MS2 spectra were acquired with a maximum ion injection time of 54 ms for increased sensitivity. For fractionated samples, half of each fraction was injected for analysis and separated over the course of 72 min (90 min total method time) with the same MS settings as for the dilution curve. Only the CIF‐fractionation was analyzed with a longer method (102 min gradient,120 min method) to ensure an equal overall LC‐MSMS analysis for all fractionation experiments. For the plasma samples, “theoretical” 500 ng peptides were injected for the single‐shot measurements. For the fractionated samples, 1/8 of each fraction was injected.

For the DIA measurements of the unfractionated samples, MS1 scans were acquired at a resolution of 120,000, the AGC target was set to 3e6 ions and the maximum ion injection time was 45 ms. MS2 spectra were acquired for 47 DIA windows after each MS1 spectrum at a resolution of 30,000. The DIA acquisition covered a mass range of 400–1000 m/z using windows of a variable width. Windows overlapped by 1 m/z. The AGC target was set to 1e6 and the maximum ion injection time was 54 ms.

### 4.2 Data Analysis

Peptide and protein identification and quantification of the DDA data was performed with MaxQuant (version 1.6.3.3) by searching against the human proteome FASTA file from Uniprot (downloaded on Feb. 2nd, 2021, 75,777 entries). Trypsin/P was set as the protease and up to 2 missed cleavage sites were allowed. Carbamidomethylation(C) was set as a fixed modification, while Oxidation(M) and Acetylation(N‐terminus) were set as variable modifications. Only for the fractionated samples match‐between‐runs was enabled. The data was further analyzed and visualized using R and RStudio. The grand average of hydropathy (GRAVY) value of peptides was calculated using the R package “Peptides” (v. 2.4.5) according to Kyte and Doolittle [45].

Peptide and protein identification and quantification of the DIA data was performed with Spectronaut (version 20.3.251119.92449) by searching against the human proteome FASTA file from Uniprot (downloaded on Jan. 21st, 2025, 20,650 entries). Trypsin/P was set as the protease and up to 2 missed cleavage sites were allowed. Carbamidomethylation(C) was set as a fixed modification, while Oxidation(M) and Acetylation(N‐terminus) were set as variable modifications. Cross‐run normalization disabled. For the analysis of the PTMs, a PTM Probing Search was performed, with maximum 3 variable modifications, Carbamidomethylation(C) was set as a fixed modification, while Oxidation(M) and Acetylation(N‐terminus) were set as known variable modifications, as probe variable modifications, Acetyl (K), Biotin, Carbamylation (KR), Carboxy, Carboxymethyl, Carboxymethyl (Any N‐term), Cation:Mg[II], Cation:Na (DE), Crotonyl, Cysteinyl, Didehydro, Dimethyl (KR), Dioxidation (MW), Formyl, Glu‐>pyro‐Glu, GlyGly (K), Hex (K), HexNAc (ST), Hydroxymethyl, Methyl (E), Methyl (KR), Myristoyl, Nitrosyl, Oxidation (M), Oxidation (P), Phospho (STY), Phosphoadenosine, Propionamide, Sulfo (STY), Trimethyl (K), Trioxidation (C) were searched.

## 5 Concluding Remarks

Here, we performed a comparative evaluation of in‐gel and SP3 sample preparation methodologies for proteomic analysis and unveiled substantial advantages, however, also disadvantages of either method. In‐gel sample preparation, renowned for its historical precedence, robustness, and compatibility with harsh lysis conditions, remains a cornerstone technique in the field. The emergence of newer methodologies, such as SP3 has led to a paradigm shift in recent years. In scenarios where sample quantities are limited, SP3 emerges as the clear frontrunner, showing overall higher sensitivity. Furthermore, SP3 appeared to be advantageous for recovering proteins associated with ribosomes and mitochondrial membranes. Still, our findings underscore the enduring relevance of in‐gel, as it yields comparable results in terms of raw protein and peptide identifications, and has its strengths particularly for specialized applications such as the identification of small proteins within complex mixtures, as evidenced by our analysis of plasma samples. In summation, SP3 is a state‐of‐the‐art proteomics method and offers more efficient protein recovery as well as higher sensitivity and reproducibility for low‐input samples. The relevance of in‐gel digestion, however, persists for specialized investigations, affirming its continued value within the proteomics toolkit of a laboratory and highlighting the importance of adapting the sample preparation method to a scientific question. However, SP3 is clearly the method of choice for modern high‐throughput automation workflows. Our findings should encourage scientists and core research infrastructures to transition their routine full proteome analysis pipelines to SP3 based workflows when in need of a robust, scalable and sensitive workflow.

## Conflicts of Interest

The authors declare no conflicts of interest.

## Associated Data

The mass spectrometry proteomics data have been deposited to the ProteomeXchange Consortium via the PRIDE [47] partner repository with the dataset identifier PXD064954.

Reviewer Access Details

Log in to the PRIDE website using the following details:

Project Accession: PXD064954

Token: ptXahDdZ40kf

## Supporting information




**Supporting File 1**: pmic70123‐sup‐0001‐FigureS1.png.

**Supporting File 2**: pmic70123‐sup‐0002‐FigureS2.png.

**Supporting File 3**: pmic70123‐sup‐0003‐FigureS3.png.

**Supporting File 4**: pmic70123‐sup‐0004‐FigureS4.png.

## Data Availability

The data that support the findings of this study are openly available in PRIDE at https://www.ebi.ac.uk/pride/, reference number PXD064954.
