# Supplementary figures and images for: Comparison of In‐Gel and SP3 Based Sample Preparation Protocols for LC‐MS/MS Based Proteomics
Source: Proteomics. 2026 Mar 29;26(7):6–16. doi: 10.1002/pmic.70123 (PMC13327715; doi:10.1002/pmic.70123)

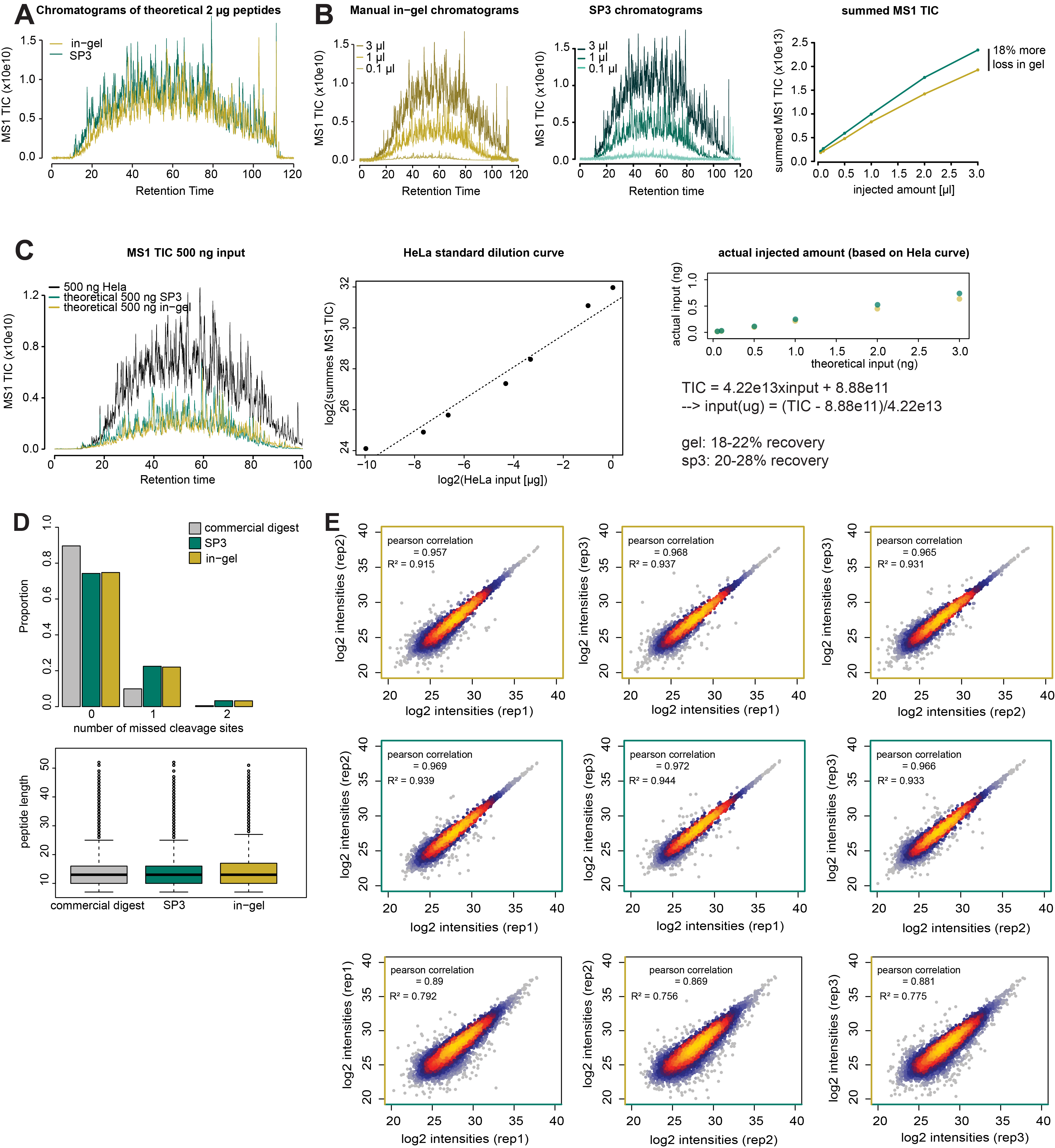

Supplement: Supplementary file 1 — Supporting File 1: pmic70123‐sup‐0001‐FigureS1.png. [file PMIC-26--s001.png]

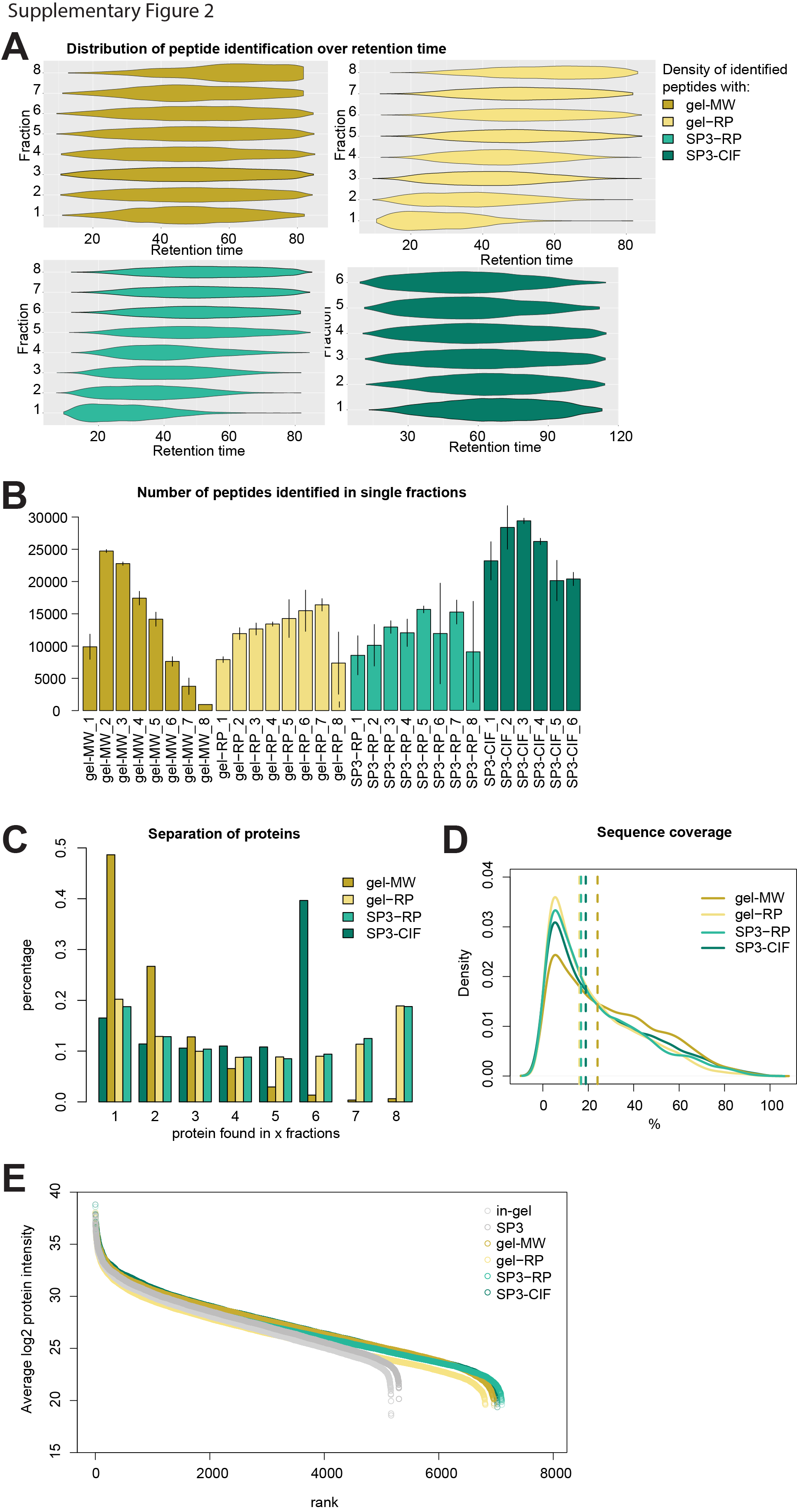

Supplement: Supplementary file 2 — Supporting File 2: pmic70123‐sup‐0002‐FigureS2.png. [file PMIC-26--s004.png]

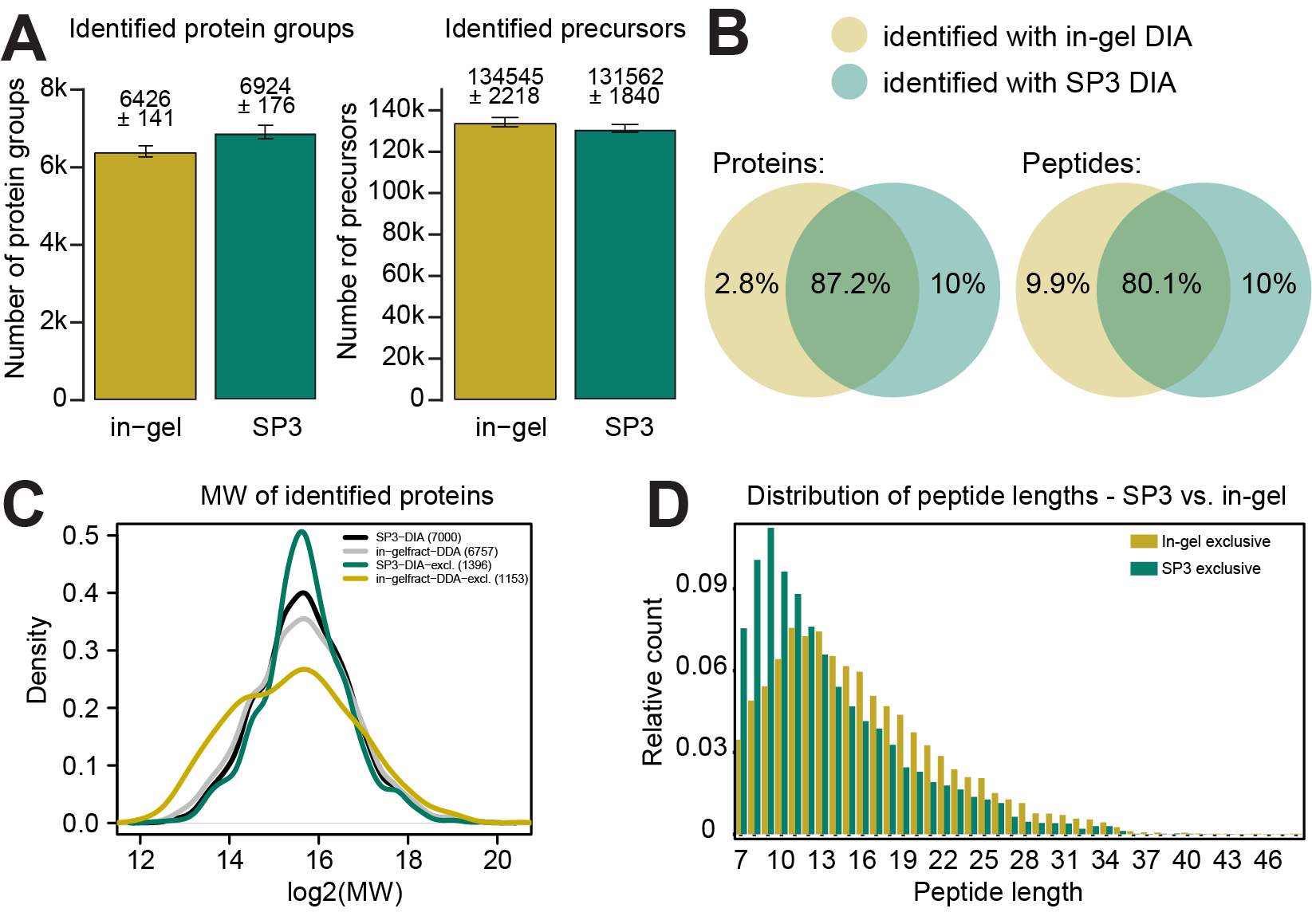

Supplement: Supplementary file 3 — Supporting File 3: pmic70123‐sup‐0003‐FigureS3.png. [file PMIC-26--s003.png]

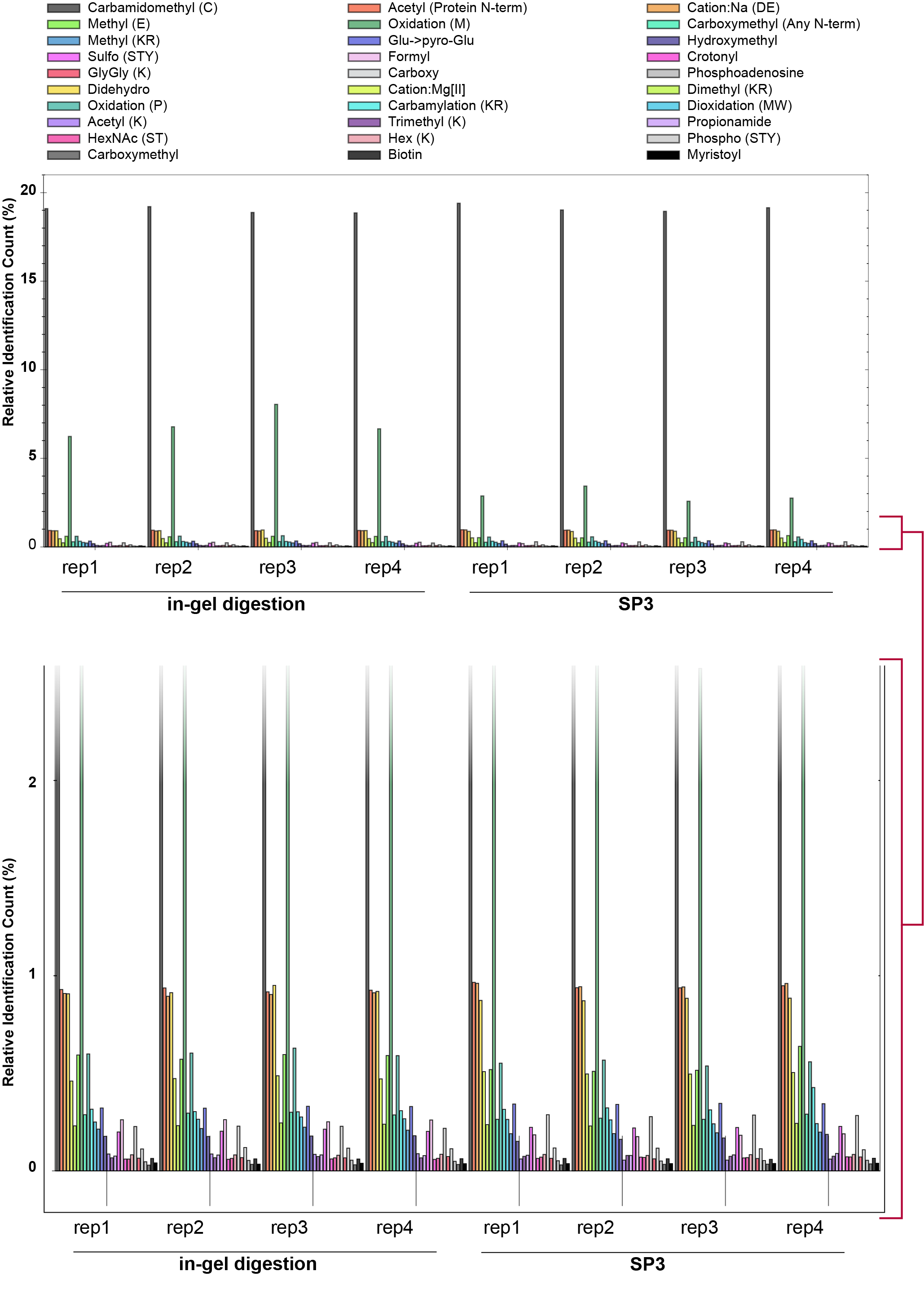

Supplement: Supplementary file 4 — Supporting File 4: pmic70123‐sup‐0004‐FigureS4.png. [file PMIC-26--s002.png]
